# Oligodendrocyte Precursor Cells Transplantation Improves Stroke Recovery *via* Oligodendrogenesis, Neurite Growth and Synaptogenesis

**DOI:** 10.14336/AD.2021.0416

**Published:** 2021-12-01

**Authors:** Wanlu Li, Tingting He, Rubing Shi, Yaying Song, Liping Wang, Zhijun Zhang, Yaohui Tang, Guo-Yuan Yang, Yongting Wang

**Affiliations:** ^1^School of Biomedical Engineering and Med-X Research Institute, Shanghai Jiao Tong University, Shanghai, China.; ^2^Department of Neurology, Ruijin Hospital, School of Medicine, Shanghai Jiao Tong University, Shanghai, China.; ^3^Department of Neurology, Zhongshan Hospital, Fudan University, Shanghai, China.

**Keywords:** OPCs transplantation, ischemic stroke, oligodendrogenesis, synaptogenesis, C-X-C motif chemokine 12, Netrin-1

## Abstract

Ischemic-induced white matter injury is strongly correlated with the poor neurological outcomes in stroke patients. The transplantation of oligodendrocyte precursor cells (OPCs) is an effective candidate for enhancing re-myelination in congenitally dysmyelinated brain and spinal cord. Nevertheless, mechanisms governing the recovery of white matter and axon after OPCs transplantation are incompletely understood in ischemic stroke. In this study, OPCs were transplanted into the ischemic brain at 7 days after transient middle cerebral artery occlusion (tMCAO). We observed improved behavior recovery and reduced brain atrophy volume at 28 days after OPCs transplantation. Moreover, our results identified that myelin sheath integrity and endogenous OPCs proliferation and migration were promoted after OPCs transplantation. By contrast, AMD3100, an antagonist of C-X-C chemokine receptor type 4, eliminated the beneficial effects of OPCs transplantation on white matter integrity and endogenous oligodendrogenesis. In addition, the improvement of neurite growth and synaptogenesis after OPCs transplantation in ischemic brain or OPC co-cultured neurons, potentially through the upregulation of Netrin-1, was indicated by increased protein levels of synaptophysin and postsynaptic density protein 95. Knockdown of Deleted in Colorectal Carcinoma, a receptor of Netrin-1, prevented increased neurite growth and synaptogenesis in neurons co-cultured with OPCs. In conclusion, our studies suggested that engrafted OPCs promoted the recovery after ischemic stroke by enhancing endogenous oligodendrogenesis, neurite growth, and synaptogenesis; the last two being mediated by the Netrin-1/DCC axis.

Ischemic stroke results in damage to the brain white matter that is comprised of myelinated axons and glia cells. Oligodendrocytes, the main producer of myelin, are vulnerable to the ischemic condition [1]. Injured oligodendrocytes can be replaced through oligodendrogenesis, which is a process that involves the proliferation, migration, and differentiation of oligodendrocyte precursor cells (OPCs) [2]. In the pathology of ischemic stroke, endogenous oligodendrogenesis is induced, but only a small amount of OPCs is recruited to the demyelinated area and differentiates into mature oligodendrocytes [3]. Therefore, methods to promote endogenous oligodendrogenesis is key to the enhancement of white matter repair after cerebral ischemia.

Stem cell therapy has emerged as a promising strategy for the treatment of cerebral ischemia. To date, adult stem cells such as bone marrow-derived mesenchymal stem cells, neural stem cells, and umbilical cord blood stem cells have been studied for their potential to treat ischemic stroke [4]. Considerable research efforts have been devoted to investigating the clinical applications of stem cell therapy on ischemic stroke. In a recent phase 1/2a study, the safety and clinical outcomes of modified bone marrow-derived mesenchymal stem cells (SB623) transplantation was evaluated in 18 patients with chronic stroke. The results showed that SB623 cells transplantation was safe in patients and the NIHSS score was improved at 1 month after transplantation. The enhancement of end points at 12 months was verified by the area of magnetic resonance T2 fluid-attenuated inversion recovery [5]. Another phase 2 trial evaluated allogeneic bone marrow-derived multipotent adult progenitor cell (MultiStem) transplantation in 126 patients. The results demonstrated both safety and efficacy of the ischemic stroke treatment [6, 7]. Meanwhile, a 300-patient phase 3 trial (MASTERS-2) is on-going [8]. The safety of stem cell transplantation in stroke patient has been confirmed in several stem cell therapeutics, yet further effort is required to understand and evaluate the clinical outcomes [9, 10].

Trophic factors secreted by transplanted cells are recognized as the primary mechanisms through which the transplanted cells facilitate oligodendrogenesis [11, 12]. White matter integrity was partially restored after adipose-derived mesenchymal stem cells administration in the subcortical stroke model, which was mediated by the molecular factors related to the axonal sprouting, remyelination and oligodendrogenesis [13]. However, these stem cells are limited by their capacity of producing oligodendrocytes. Transplantation of OPCs directly provides a viable source for the oligodendrocytes generation and white matter repairing. A large number of OPCs exists in newborn and adult brains of rodent and human [14]. Recently, it has been reported that the transplantation of fetal or adult OPCs, OPCs derived from embryonic stem cells or human induced pluripotent stem cells, boosted myelination in congenitally dysmyelinated brain and spinal cord [15-17]. Our group have previously demonstrated that administrating OPCs immediately after cerebral ischemia effectively protected the blood-brain barrier (BBB) through the Wnt/β-catenin pathway in a mouse model [18]. However, whether and how delayed OPCs transplantation affect oligodendrogenesis and myelination, as well as neurite growth and synaptogenesis, have not yet been investigated in ischemic stroke models.

In this work, we investigated the effects of delayed OPCs transplantation on post-stroke recovery using a mouse transient middle cerebral artery occlusion (tMCAO) model. OPCs were isolated from the cerebral cortex of newborn rats and then stereotactically injected into the perifocal area at 7 days after tMCAO. We aim to explore whether delayed OPCs transplantation promotes the functional recovery of ischemic mice, focusing on its influences on oligodendrogenesis, neurite growth and synaptogenesis.

## MATERIALS AND METHODS

### Ischemic stroke model

All protocols for animal experiments were approved by the Institutional Animal Care and Use Committee of Shanghai Jiao Tong University. Adult male C57BL/6 mice, aged 10-12 weeks, weighing 20-25 grams were housed in an SPF vivarium under a 12-hours dark-light cycle. Mice were anesthetized with 1.5% isoflurane in oxygen/nitrous oxide (30%/70%) and placed on a heating pad (RWD Life Science, Shenzhen, China) throughout the surgery to maintain the body temperature at 37 °C. A silicon coated 6-0 suture (Covidien, Mansfield, MA) was gently inserted into the internal carotid artery through an incision in the external carotid artery until the suture tip reached the bifurcation of middle cerebral artery. Cerebral blood flow was measured using Laser Doppler Flowmetry (Moor Instruments, Devon, UK) to confirm the occlusion of the MCA. A reduction of cerebral blood flow down to below 15% of baseline was considered as successful occlusion. The suture was withdrawn at 1 hour after occlusion to allow reperfusion. A cerebral blood flow recovery back to over 80% of baseline was regarded as successful reperfusion [19, 20].

### Animals grouping

Animals in the sham group (n=8) underwent anesthesia, midline incision on the neck, and isolation of the left common carotid artery, the external carotid artery and the internal carotid artery without insertion of a suture to occlude the MCA. For the other three groups, animals (n=51, death rate after tMCAO within 7 days was 20.3%) were randomly assigned into three groups at 7 days after tMCAO and before the OPCs transplantation. 13 animals were assigned to the normal saline vehicle group (NS), 25 were to the OPCs transplantation group (OPC) including 12 animals for the OPCs tracking experiment, and 13 to the OPC and AMD co-administration group (OPC+AMD).

### OPCs isolation, characterization, and transplantation

OPCs were isolated from the cerebral cortex of newborn Sprague-Dawley rat pups. The cerebral cortex was separated, followed by the mechanical dispersing and chemical digestion, and then the single-cell suspension was seeded on cell culture flasks coated with poly-D-lysine (Sigma-Aldrich, MO, USA). After being cultured as mixed glia for 7-10 days in DMEM with 10% fetal bovine serum (Gibco, NY, USA), OPCs were harvested by shaking at 200?rpm on a shaker platform.

OPCs was transplanted through stereotactic injection at 7 days after tMCAO. Five microliters of normal saline containing 1×10^6^ OPCs or normal saline alone was injected into one site of perifocal area with the following coordinate: Anterior Posterior (AP) = 0 mm, Medial Lateral (ML) = -2 mm, and Dorsal Ventral (DV) = -2.5 mm.

In the OPC and AMD3100 co-administration group, C-X-C chemokine receptor type 4 (CXCR4) antagonist AMD3100 (1 mg/kg, Sigma-Aldrich, Darmstadt, Germany) was injected intraperitoneally daily after OPCs transplantation until animal sacrifice.

### OPC labeling with carboxyfluorescein diacetate succinimidyl ester (CFDA-SE) and quantification

To trace the transplanted OPCs, isolated OPCs were stained with CFDA-SE before transplantation. OPCs were suspended with CFDA-SE staining solution (10 μM) and incubated at 37 °C for 15 mins. After centrifugation, OPCs was separated from staining solution and incubated in medium for another 30 mins followed by a final wash step with medium prior to transplantation.

To trace and quantify the labeled cells after transplantation, the brain slices were obtained at 7, 14, 35, and 70 days after tMCAO and stained with Neuron-glial antigen 2 (NG2)/DAPI, Myelin basic protein (MBP)/DAPI, Glial fibrillary acidic protein (GFAP)/DAPI, or Iba-1/DAPI. The sum of the CFDA-SE fluorescence (green) for all pixels (*F_C_*) was acquired using ImageJ, and it was divided by the pixel numbers of each nucleus (*P_n_*) to yield the average pixels for each CFDA-SE cell (*P_C_*). Finally, the total number of CFDA-SE cells (*N_C_*) was calculated by dividing *F_C_* with *P_C_*.

### Neurological Behavior Test

Functional recovery in motor behavior was assessed by the rotarod test and hanging wire test by a researcher blinded to the animal treatment assignment. Functional cognitive recovery was assessed using the passive avoidance and T-maze tests by a researcher blinded to the animal treatment assignment.

In the rotarod test, mice were first adapted for 1 min on the rod then ran for 5 mins while the rod was accelerated to 20 rpm. The rod was then accelerated to 40 rpm over 5 mins and the time that the mice maintained on the rod since 20 rpm was recorded [21]. In the hanging wire test, mice were hung on a horizontal wire. The wire is 1.6?mm in diameter, 50?cm in length, and elevated at 30?cm above the floor. We scored the mice based on the number of times they reach the terminal (earn one point) and the number of falls (loss one point) in 180 s. The average of three tests was used for analysis for both the rotarod test and hanging wire test.

The passive avoidance task is used to evaluate learning and memory in animals. Mice were put into a cage composed of a light chamber and a dark chamber, and a foot-shock was delivered through a grid on the cage floor every time they entered the dark chamber. 24 hours later, mice were placed in the same cage with the light chamber and dark chamber without a foot-shock trigger. Mice with healthy cognitive ability and memory tend to avoid entering the dark chamber where they had been shocked. We recorded the latency of entering the dark zone in 10 mins as a parameter to assess cognitive ability and memory. T-maze aims to test the willingness of animals to explore a new environment. Briefly, mice were placed in the start arm and were allowed to choose the left or the right arm at will. They were then separated in the arm they first entered and allowed to explore for 1 min. The choices of arms made by the mice in the next 11 continuous tests were recorded. Each spontaneous alteration of entering different arms between two consecutive tests was counted towards final alteration percentage.

### Assessment of brain lesion size

Mice were euthanized and transcardially perfused with normal saline and 4% paraformaldehyde (Sinopharm Chemical Reagent, Shanghai, China). Brains were removed immediately after perfusion and were post-fixed with 4% paraformaldehyde for 4 hours and soaked in 30% sucrose overnight. The sucrose cryoprotected brains were then frozen at -80? and sectioned into 30-μm thick floating coronal sections with a cryostat (Leica, Solms, Germany). The collected slices were stored in antigen protective solution (50% PBS, 20% glycol, and 30% glycerol) at -20?. A total of 10 coronal sections spanning the entire injured area were mounted on glass slides and stained with cresyl violet to evaluate brain atrophy volume. The results were measured by ImageJ software (National Institutes of Health, Bethesda, MD) and calculated with the following formulas:

$$V(\mathrm{atrophy})=?\frac{h}{3}\left[{?S}_{n}+\sqrt[{}]{?S_{n}?S_{n+1}}+?S_{n+1}\right]{?S}_{n}=S_{n_{\mathrm{Contl}}}-S_{n_{\mathrm{Ipsil}}}{?S}_{n+1}=S_{{n+1}_{\mathrm{Contl}}}-S_{{n+1}_{\mathrm{Ipsil}}}$$

*ΔS* were calculated by subtracting the normal area of the ipsilateral hemisphere from the contralateral hemisphere area. *ΔS_n_* and *ΔS_n+1_* represent the infarct areas of two adjacent sections analyzed. *h* indicates the distance between two adjacent coronal sections, *h* = 300 μm.

### 5-Bromo-2′-deoxyuridine (BrdU) injection

BrdU (50 mg/kg, Sigma-Aldrich, St. Louis, MO) was intraperitoneally injected daily from 7 to 35 days after tMCAO.

### Culture of primary neurons and co-culture of neurons and OPCs

Primary neurons were isolated from the cerebral cortex of newborn Sprague-Dawley rats. The isolated cells were seeded into 6-well plates pre-coated with poly-D-lysine at 6×10^5^ cells/well. After 3 or 6 days of culturing the neurons, the co-culture system was established by seeding OPCs in Transwell with 0.4-µm pore size (Millipore, Darmstadt, Germany). Neurite growth was measured using the neurons cultured for 9 or 12 days, and synaptogenesis was assessed in neurons cultured for 12 days. The co-culture time points were chosen based on the reported properties of cultured neurons *in vitro* [22]. It has been reported that the extended neurites of cortical neurons were observed after 5-days culturing, but only a few expressions of synaptic markers at 5 days. It showed increased expression of synaptophysin with neuron maturation until the remarkable synaptogenesis occurred at 15 days of culturing. Moreover, extensive culture of cortical neurons (over 20 days) *in vitro* results in a complex profile of protein damage. In general, neurons cultured for 10 to 15 days are widely applied to the synaptogenesis assessment [23-25].

### Deleted in colorectal carcinoma (DCC) siRNA interference

The sequence of DCC-siRNA used in this work was 5’-GCAAUUUGCUCAUCUCUAATT-3’. The siRNA transfection was performed as previously reported, in which the negative control was a random non-targeting siRNA of the same length [26]. Briefly, neurons were transfected with 20-nM siRNA (Genepharma, Shanghai, China) premixed with 4-µl Lipofectamine® 2000 (Invitrogen) and 100-µl Opti-MEMI (Gibco). The total RNA was extracted from neurons at 24 hours after transfection, and proteins were obtained at 48?hours after transfection to test the interference efficiency of siRNA. In co-culture experiments, siRNA was added into the medium throughout the co-culture period.

### Western blot analysis

Mice were sacrificed at 35 days after tMCAO. Brain was quickly removed and cut into 4 sections with 2-mm thickness. The protein of ipsilateral brain was extracted from the ipsilateral subcortical area of the second rostral section including striatum and corpus callosum. These regions are selected because the white matter and axons are enriched in these regions, which are also areas vulnerable to the ischemic injury [27-29]. For *in vitro* experiments, proteins of neurons were collected from cultured neurons on day 9 and day 12. In detail, the extracted proteins were quantified using a BCA kit (Beyotime, China). A total of 40-μg protein were loaded into a 10% SDS-PAGE gel for electrophoresis, and then the proteins in the gel were transferred onto a polyvinylidene fluoride membrane. After being blocked with 5% milk, the membrane was incubated with primary antibodies at 4 ? overnight, washed, and incubated with HRP-conjugated secondary antibodies for 2 hours at room temperature. Finally, target proteins on the membrane were visualized by reacting with an enhanced chemiluminescence substrate (Pierce, Rockford, IL) and imaged using an imaging system (Bio-Rad, Hercules, CA). The results were analyzed using ImageJ software.

### Immunofluorescence staining

Brain floating slices were permeabilized in 0.3% Triton X-100 (Sigma-Aldrich, St. Louis, MO) for 10 mins. After being blocked with 10% bovine serum albumin (Yeasen, Shanghai, China), floating slices were incubated with primary antibodies overnight at 4?, followed by incubation with secondary antibodies for 1 hour at room temperature. We captured images with a confocal microscope (Leica, Solms, Germany), and analyzed the images with ImageJ software. For NG2/BrdU double immunostaining, floating slices were first permeabilized with 0.3% Triton X-100 for 20 mins, followed by incubation with 2 mol/L HCl for 30 mins at 37 °C and HCl neutralized with sodium tetraborate. Brain slices were then blocked with 10% bovine serum albumin, incubated with primary antibodies overnight at 4?, washed, and reacted with fluorescently labeled secondary antibodies for 1 hour. Antibodies information can be found in the Supplementary Information.

### Luxol fast blue (LFB) staining

Brain sections were immersed in a 0.1% ethanol solution containing LFB (Sigma-Aldrich, St. Louis, MO) at 37? overnight and then washed with distilled water. Sections were then incubated in 0.01% lithium carbonate until the color was developed. Finally, brain sections were dehydrated through graded alcohols and imaged using light microscopy. The integrated optical density (IOD) of LFB was measured by ImageJ software in the corpus callosum (CC) and striatum regions.

### Image collections of staining results

To quantify the IOD of Microtubule-associated protein 2 (MAP2) and Synaptophysin, 3 fields were randomly selected along the perifocal region, and 3 sections spaced 2 mm apart were sampled for every mouse.

For quantifications of NG2 and BrdU double-positive cells at the subventricular zone (SVZ), or only NG2 positive cells at perifocal areas, three random fields along the SVZ were photographed for each brain section, and 3 sections covered 1 mm were analyzed for every mouse.

The white matter evaluation was conducted using MBP staining and LFB staining. The IOD of MBP was quantified with one field including the striatum and corpus callosum, and 3 sections spaced 1 mm were sampled for every mouse. The IOD of LFB staining was measured by ImageJ software in the corpus callosum with the images captured by light microscopy. Three fields were randomly selected within the corpus callosum region, and 3 sections spaced 2 mm were sampled for every mouse.

### Statistical Analysis

All results were presented as means ± SEMs. All statistical analysis was carried out with SPSS v21.0 (SPSS Inc., Chicago, IL) using one-way analysis of variance (ANOVA) followed by Two-tailed Student’s *t*-test or Tukey's Honest Significant Difference test, except for the non-parametric analysis was used for analyzing the results of T-maze test. A value of *p* <0.05 was considered statistically significant.

## RESULTS

### OPCs reduced brain atrophy volume and promoted functional recovery of cerebral ischemic mice

The experimental scheme is shown in Supplementary Figures 1 in the Supplementary Information. We transplanted OPCs into the perifocal area of mice brain at 7 days after tMCAO. Prior to the transplantation, immunostaining analysis of the isolated OPCs showed that 88% of isolated cells were positive for both NG2 and PDGFR-α (Supplementary Fig. 2), indicating that the majority of transplanted cells were OPCs.

**Figure 1.**
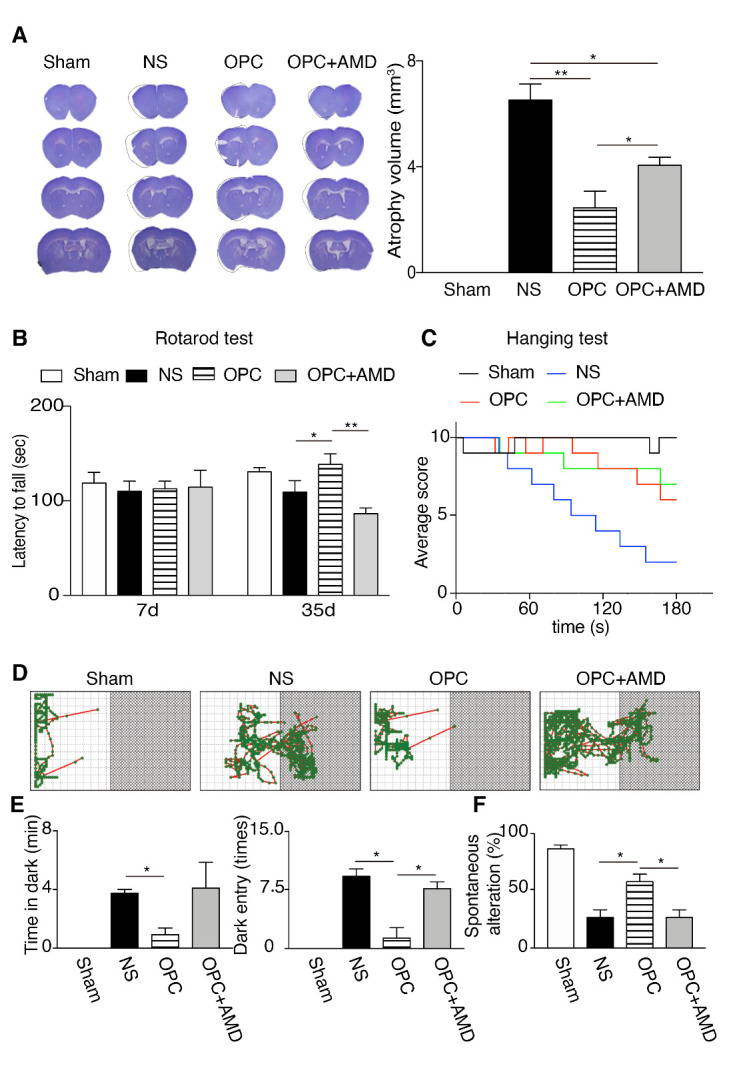
OPCs transplantation at 7 days after tMCAO reduced brain atrophy volume and promoted functional recovery of mice at 5 weeks after tMCAO. (A) Representative photos and quantification result of brain coronal sections stained with cresyl violet at 5 weeks after tMCAO. Sham (n=5), NS (n=8), OPC (n=8), and OPC+AMD (n=8). The black lines in the photos are the tracing of the contralateral hemisphere onto the ischemic hemisphere. (B) The result of rotarod test at 7 days and 35 days after tMCAO. Sham (n=8), NS (n=13), OPC (n=13), OPC+AMD3100 (n=13). (C) The result of hanging wire test at 35 days after tMCAO. Sham (n=8), NS (n=13), OPC (n=13), OPC+AMD3100 (n=13). D, The locomotion trajectories of mice for the passive avoidance test. (E) The time in dark zone and the numbers of dark-zone entering were summarized in the bar graphs in Sham (n=8), NS (n=13), OPC (n=13), OPC+AMD (n=13) groups. (F) The result of T-maze test in Sham (n=8), NS (n=13), OPC (n=13), OPC+AMD (n=13) groups. Data are presented as means ± SEMs, *p <0.05, **p <0.01. NS: tMCAO mice receiving normal saline, OPC: tMCAO mice receiving OPCs transplantation, OPC+AMD: tMCAO mice receiving OPCs and AMD3100.

To investigate the effects of OPCs transplantation in brain atrophy and behavior outcomes, we evaluated atrophy volume and conducted behavior tests at 5 weeks after tMCAO. The results illustrated that OPCs transplantation reduced brain atrophy volume compared with the NS group. Additionally, co-administration of AMD3100 partially eliminated the positive influence of OPCs on the atrophy volume (Fig. 1A; *p* (NS vs OPC) = 0.006, *p* (NS vs OPC+AMD) = 0.047, *p* (OPC vs OPC+AMD) = 0.041). Rotarod test and hanging wire test were performed to assess motor function and strength at 5 weeks after stroke, as previous studies reported [21, 30, 31]. All animal performance was evaluated comparably in the rotarod test before OPCs injection at 1 week after tMCAO. Mice received OPCs transplantation exhibited improved rotarod outcome compared with the NS mice at 35 days after tMCAO, and the AMD3100 group performed worse than the OPCs group (Fig. 1B; *p* (NS vs OPC at 35 days) = 0.043, *p* (OPC vs OPC+AMD at 35 days) = 0.003). Administration of OPCs promoted the outcome of the hanging wire test compared with NS mice, whereas no significant difference was observed in the hanging wire test between the OPC group and the OPC + AMD3100 group (Fig. 1C).

Passive avoidance and T-maze tests were applied to identify the functional recovery of cognitive ability. By comparing the results of sham group with stroke group, longer time in the dark zone was observed in the stroke animals. Meanwhile, OPCs group presented decreased time in dark zone and reduced numbers of entering the dark zone compared with NS group, and this improvement was abolished through the AMD3100 administration (Fig. 1D-E; Time in dark zone, *p* (NS vs OPC) = 0.020; Numbers of dark zone entry, *p* (NS vs OPC) = 0.014, *p* (OPC vs OPC+AMD) = 0.017). In the T-maze test, normal mice chose the different arm in the second test during two consecutive tests. Mice injected with OPCs exhibited higher rate of spontaneous alteration than the NS vehicle control mice, while co-administration of AMD3100 abolished this improvement (Fig. 1F; *p* (NS vs OPC) = 0.021, *p* (OPC vs OPC+AMD) = 0.021). These results indicated that OPCs transplantation at the recovery phase reduced brain atrophy and facilitated both motor and cognitive functional recovery of cerebral ischemic mice.

**Figure 2.**
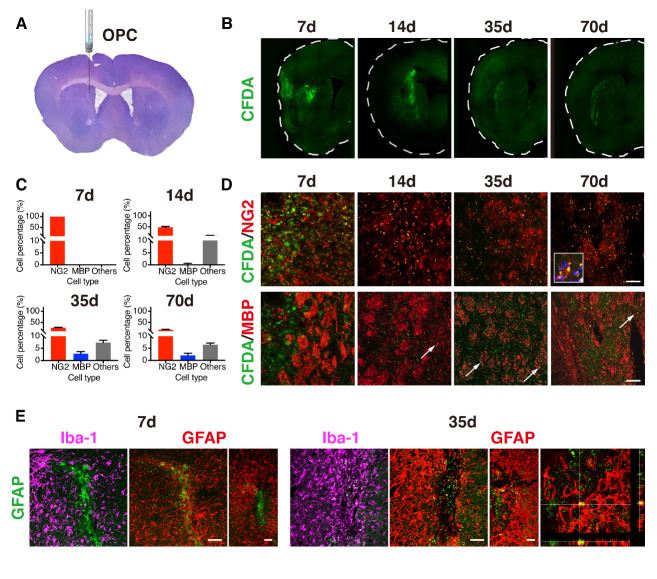
The distribution and differentiation of transplanted OPCs in the ischemic brain. (A) The photo of brain coronal section stained with cresyl violet at 5 weeks after tMCAO illustrates the injection position of OPCs. (B) Photomicrographs of ipsilateral hemispheres showing the distributions of transplanted OPCs labeled with CFDA-SE (green) at 7, 14, 35, 70 days after tMCAO. (C-D) Enlarged images showing NG2^+^/CFDA^+^ or MBP^+^/CFDA^+^ cells in the injection sites that are indicated in the (A) with the red dotted line. The bar graph summarizing the quantification of cell distributions (n=3/time point). Arrows indicating MBP^+^/CFDA^+^ cells. (E) Photomicrographs of CFDA-SE (green) and Iba-1 (purple) or GFAP (red) in the periinfarct area at 7 and 35 days after tMCAO and after OPCs transplantation. CFDA-SE/GFAP results showing with the photomicrograph in the periinfarct area, in the larger region, and the enlarged image. Scale bar, 100 μm. CFDA-SE: carboxyfluorescein diacetate succinimidyl ester.

### The differentiation of transplanted OPCs were limited in the perifocal area of ischemic brain

The transplanted OPCs were labeled with CDFA-SE to trace cell fates when injected into the perifocal area at 7 days after tMCAO (Fig. 2A). The injection site, AP = 0 mm, ML = -2 mm, and DV = -2.5 mm, was determined by the position of perifocal area in the striatum based on our previous study of imaging the injury region in the same model [32]. The labeled cells were observed in the injecting area immediately after transplantation, and then expanded to a larger field at 14 days. Yet, they died away at a slow pace up to 70 days after tMCAO (Fig. 2B-D). Only 32.5% of transplanted cells was survived after 70 days of tMCAO, including the 17.6% of NG2-positive cells remained, and the 2.0% was differentiated into MBP-positive oligodendrocytes. This suggested that transplanted OPCs had limited capacity to produce mature oligodendrocytes, implying the possibility of the essential role of trophic factors after OPCs transplantation. Given that the OPCs have the capacity of differentiating into neurons and astrocytes, in addition to oligodendrocytes, CFDA^+^/NG2^-^/MBP^-^ cells were evaluated by immunostaining against MAP2, GFAP, and Iba-1. As shown in Figures 2E and S3, the transplanted OPCs differentiated into GFAP-positive astrocytes, but not into MAP2-positive neurons. Additionally, some CFDA-SE signals could be found in Iba-1-positive microglia, indicating these transplanted OPCs were phagocyted by microglia.

**Figure 3.**
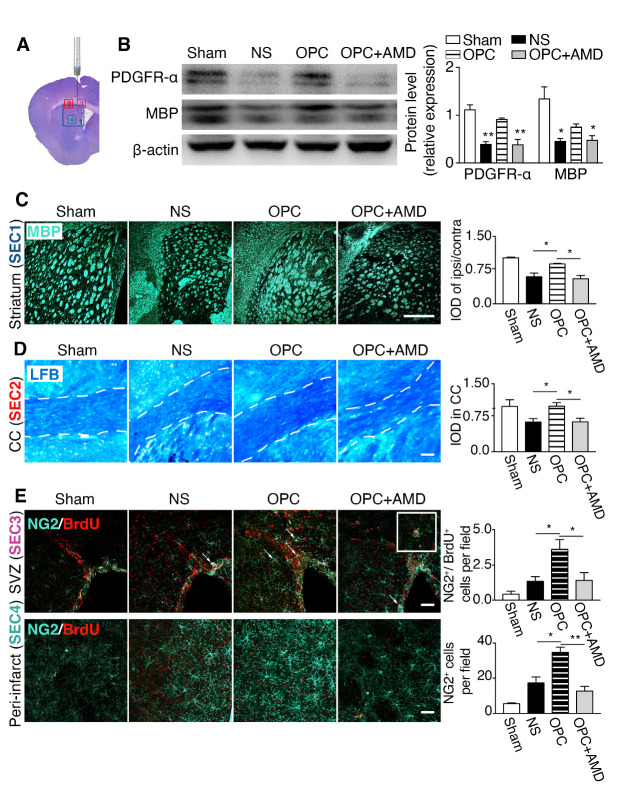
OPCs protected myelin sheath integrity and promoted the proliferation of endogenous OPCs, and migration of OPCs. (A) The ipsilateral photo of brain coronal section stained with cresyl violet at 5 weeks after tMCAO illustrates the regions of interest imaged in sections. (B) The Western blot results of PDGFR-α and MBP in the ischemic striatum (n=3/group). *p <0.05, **p <0.01 compared with the OPC group. (C) Photomicrographs of MBP (light blue) in the striatum and the quantification of MBP IOD. (D) LFB myelin staining (blue) in the corpus callosum and the quantification data (n=3/group). The boarder of corpus callosum is outlined by the dotted lines. (E) Images of NG2 (light blue) and BrdU (red) staining in the SVZ and periinfarct area of striatum. Arrows indicating NG2^+^/BrdU^+^ newborn OPCs. The inset at the right corner is the magnified image of a NG2^+^/BrdU^+^ cell. Bar graphs are quantifications of NG2^+^/BrdU^+^ cells in the SVZ and NG2^+^ OPC cells that migrated to the periinfarct area (n=3/group). Scale bar, 50 μm. Data are presented as means ± SEMs, *p <0.05, **p <0.01. IOD: integrated optical density, LFB: luxol fast blue, CC: corpus callosum, SVZ: subventricular zone.

**Figure 4.**
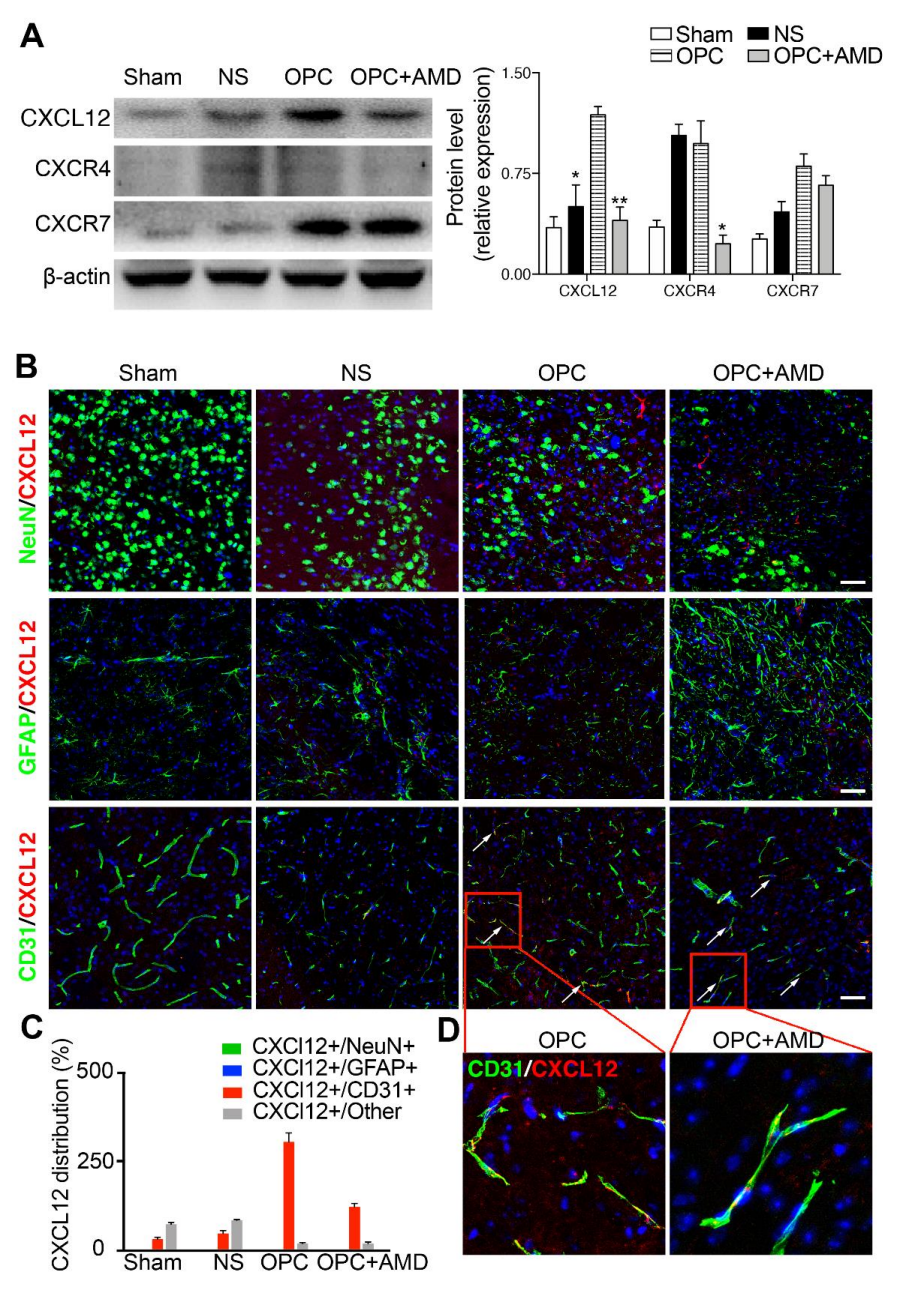
CXCL12 was upregulated after OPCs transplantation, which was abolished by the AMD3100. (A) Western blot results of CXCL12, CXCR4, and CXCR7 in the ischemic striatum (n=3/group). *p <0.05, **p <0.01 compared with the OPC group. (B) Photomicrographs of NeuN or GFAP or CD31 (green) and CXCL12 (red) in the perifocal area of ischemic mice. (C) The quantification data of CXCL12 distribution in the different types of cells. (D) Magnified image sections highlighted by the red boxes in (B). Scale bar, 50 μm. Data are presented as means ± SEMs. CXCL12: C-X-C motif chemokine 12, CXCR4: C-X-C chemokine receptor type 4, CXCR7: C-X-C chemokine receptor type 7.

### OPCs protected myelin sheath integrity and increased the endogenous OPC proliferation and migration

To investigate whether OPCs transplantation protect myelin sheath integrity, protein expression of PDGFR-α and MBP was measured with Western blot. Results showed that PDGFR-α and MBP were increased in OPC group compared with NS vehicle control and reduced in the AMD3100 co-administration group (Fig. 3B; PDGFR-α, *p* (NS vs OPC) = 0.001, *p* (OPC vs OPC+AMD) = 0.010; MBP, *p* (NS vs OPC) = 0.030, *p* (OPC vs OPC+AMD) = 0.048). MBP immunostaining and LFB staining were conducted at 5 weeks after tMCAO to further confirm these effects, which was in good consistent with the observed results from Western blot. The targeted regions of staining were illustrated in Figure 3A. The results clarified that MBP intensity was higher in OPC group than the NS group or AMD3100 group (Fig. 3C; *p* (NS vs OPC) = 0.026, *p* (OPC vs OPC+AMD) = 0.011). LFB is a common method that used to detect demyelination in brain. Higher density of myelin sheath indicated by LFB staining was observed at the CC in the OPC group compared with NS group or AMD3100 group (Fig. 3D; *p* (NS vs OPC) = 0.033, *p* (OPC vs OPC+AMD) = 0.035p). In addition, we examined the proliferation and migration of OPCs by the NG2 and BrdU immunostaining. As can be seen from Figure 3E, the number of newborn OPCs, which were positive for both NG2 and BrdU, was increased in the ipsilateral SVZ of OPC group (*p* (NS vs OPC) = 0.043, *p* (OPC vs OPC+AMD) = 0.050). Meanwhile, the number of migrating OPCs in the perifocal area, which were NG2 positive and BrdU negative, was higher in OPC group than NS group (*p* (NS vs OPC) = 0.018, *p* (OPC vs OPC+AMD) = 0.005). These data indicated that OPCs transplantation protected myelin sheath integrity, increased proliferation of OPCs at SVZ, and promoted migration of OPCs to perifocal area. Because the limited survival and differentiation of transplanted OPCs, we concluded that the endogenous source played an important role in oligodendrogenesis enhancement.

### OPCs promoted endogenous oligodendrogenesis, which was inhibited by blocking CXCR4

The mechanism involved in the promoted oligodendrogenesis after OPCs transplantation was further explored. AMD3100, an antagonist of CXCL12 receptor CXCR4, abolished the protection effects of OPC transplantation on the white matter integrity and the promotion of OPC proliferation and migration (Fig. 3E). It is therefore plausible that the transplanted OPCs promoted myeline sheath integrity, as well as OPC proliferation and migration through CXCL12/CXCR4 axis. The results of Western blot displayed that CXCL12 was upregulated in brain tissue of OPC group. In the AMD3100 co-treated group, CXCL12 and CXCR4 levels both decreased compared with OPC group (Fig. 4A; CXCL12, *p* (OPC vs OPC+AMD) = 0.001, *p* (NS vs OPC) = 0.017). Moreover, the immunostaining of CXCL12 and different cell markers were applied to detect the source of upregulated CXCL12 in the brain (Fig. 4B-D). Among neurons, astrocytes, and endothelial cells, the existence of CXCL12 could be found in the area which was positive for endothelial cell marker (CD31).

### OPCs enhanced neurite growth and synaptogenesis of ischemic stroke mice

**Figure 5.**
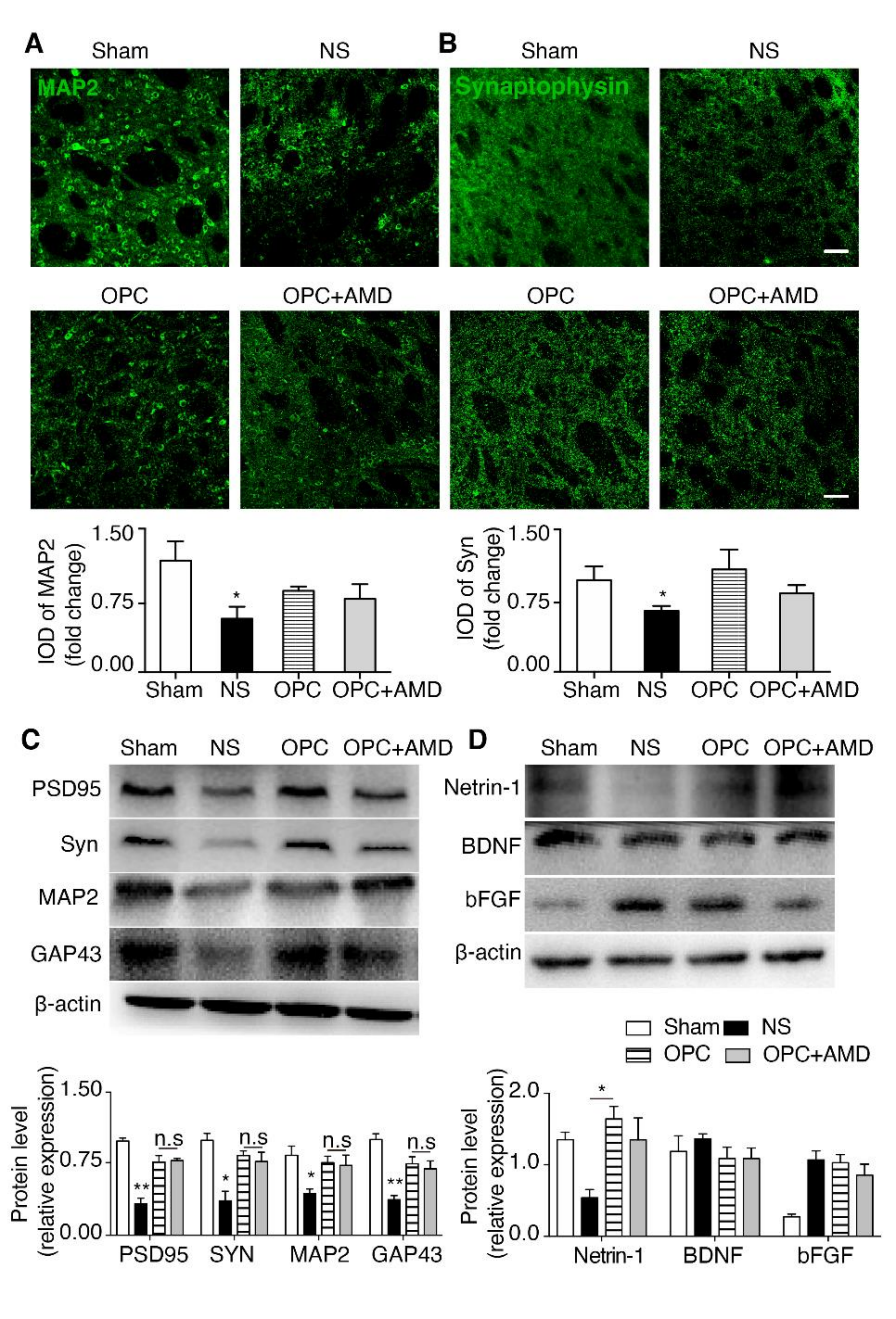
Netrin-1 upregulation after OPCs treatment was associated with increased neurite growth and synaptogenesis in ischemic stroke mice. (A) Photomicrographs and IOD quantification of MAP2 (green) in the striatum (n=3/group). *p <0.05, **p <0.01 compared with the OPC group. (B) Photomicrographs and IOD quantification of Synaptophysin (green) in the striatum (n=3/group). *p <0.05, **p <0.01 compared with the OPC group. (C) Western blot results of PSD95, Synaptophysin, MAP2 and GAP43 in ischemic striatum (n=3/group). (D) Western blot results of Netrin-1, BDNF and bFGF in ischemic striatum (n=3/group). *p <0.05, **p <0.01, n.s no significance compared with the OPC group. Scale bar, 50 μm. Data are presented as means ± SEMs. MAP2: microtubule-associated protein 2, PSD95: postsynaptic density protein 95, BDNF brain-derived neurotrophic factor, bFGF: basic fibroblast growth factor.

There is no denying that the increase of new oligodendrocytes is strongly associated with axon growth in perifocal areas, which might contribute to the functional recovery after stroke. Immunostaining and Western blot results displayed that axon related proteins (MAP2 and Growth associated protein 43 (GAP43)) and synapse related proteins (Postsynaptic density protein 95 (PSD95) and Synaptophysin) were increased after OPCs transplantation compared with NS group (Fig. 5A, *p* (NS vs OPC) = 0.045. Figure 5B, *p* (NS vs OPC) = 0.043). Figure 5C, PSD95, *p* (NS vs OPC) = 0.007; Synaptophysin, *p* (NS vs OPC) = 0.014; MAP2, *p* (NS vs OPC) = 0.0017, GAP43, *p* (NS vs OPC) = 0.001). However, AMD3100 did not inhibit the augment on neurite growth and synapse in brain tissue, which indicated that other pathways besides CXCL12/CXCR4 were likely participated in this process. We further investigated other factors that were known to be concerned with neural remodeling, including Netrin-1, brain-derived neurotrophic factor (BDNF), and basic fibroblast growth factor (bFGF). Western bot results presented that only the expression of Netrin-1 was increased in OPC group compared with NS group. (Fig. 5D; Netrin-1, *p* (NS vs OPC) = 0.006). On the basis of above results we concluded that OPCs transplantation enhanced neurite growth and synaptogenesis in cerebral ischemic mice, which was not mediated by CXCL12/CXCR4 but possibly related to the increased Netrin-1.

### OPC-conditioned medium increased neurite growth and synaptogenesis of primary neurons via Netrin-1/DCC

**Figure 6.**
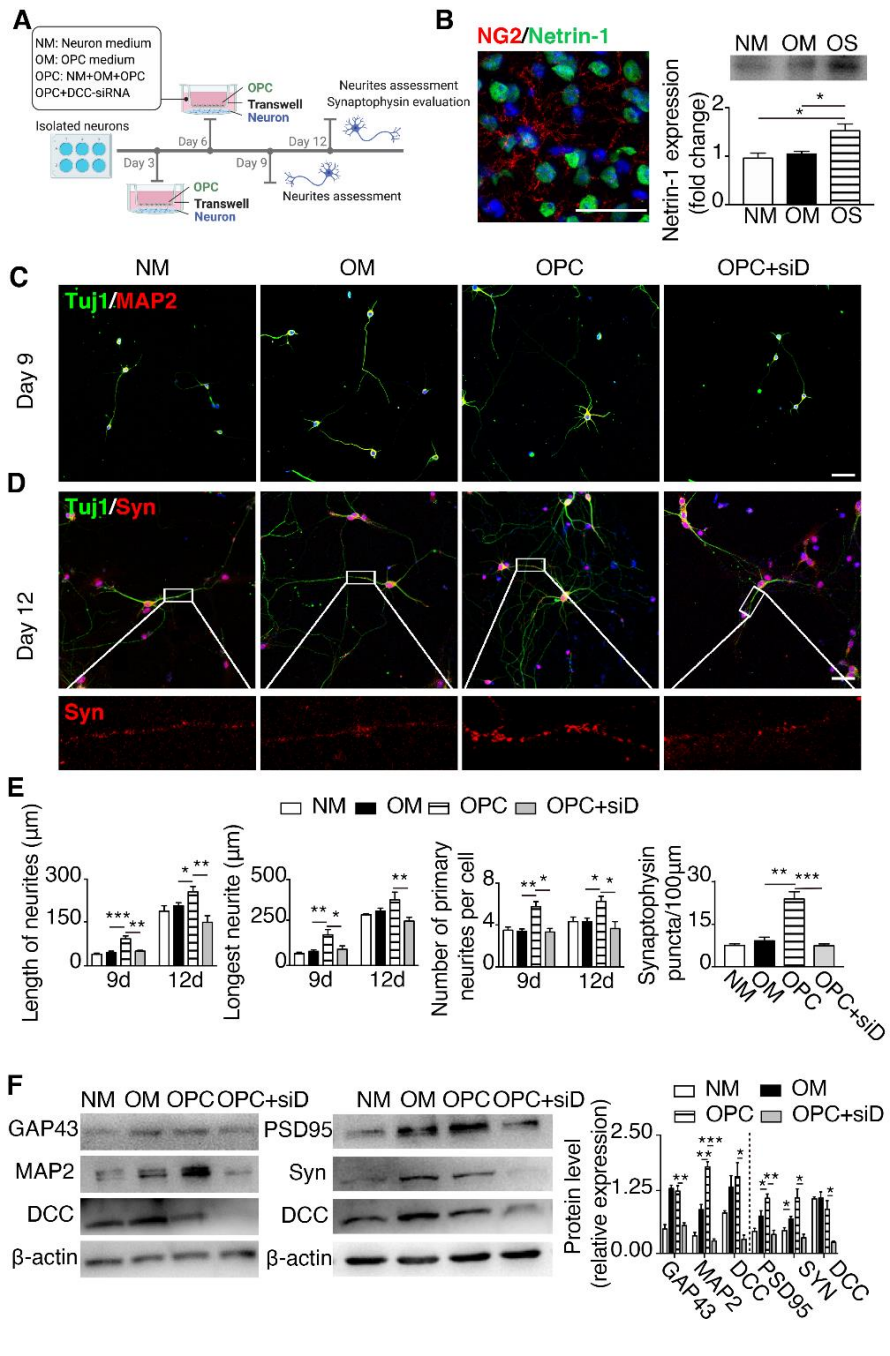
OPC-conditioned medium promoted neurite growth and synaptogenesis of primary neurons via Netrin-1/DCC. (A) The schematic illustration of co-culture system of neurons and OPCs using the Transwell system. (B) Image of Netrin-1 (green) and NG2 (red) staining in the ischemic hemisphere. The right panel showing the result of Western blot for Netrin-1 protein in the OPC supernatant. (C-D) Photomicrographs of Tuj1 (green) and MAP2 (red) staining of primary neurons cultured for 9 days, and Tuj1 (green) and Synaptophysin (red) staining of primary neurons cultured for 12 days. Magnified images of Synaptophysin (red) are shown at the bottom panel. Scale bar, 50 μm. (E) Bar graphs showing the quantifications of the average length, longest length and numbers in neurites (n=6-12/group), and the quantification of synaptophysin in primary neurons cultured for 12 days (n=6-12/group). *p <0.05, **p <0.01, ***p <0.001. (F) Western blot of MAP2, GAP43, DCC in primary neurons cultured for 9 days, and quantification data (n=3/group) and the Western blot of PSD95, Synaptophysin, and DCC in primary neurons cultured for 12 days, and quantification data (n=3/group). *p <0.05, **p <0.01 compared with the OPC group. Data are presented as means ± SEMs. Syn: synaptophysin, DCC: deleted in colorectal carcinoma, NM: neuron medium, OM: OPC medium, OPC denotes OPCs plus OPC medium, OPC+DCC-siRNA indicates DCC-siRNA transfected OPCs plus OPC medium, OS: OPC supernatant.

To further explore the influence of Netrin-1 raised by OPCs on neural remodeling, we established a co-culture system using Transwell with 0.4-μm pore size. Primary neurons were cultured on the lower chamber and OPCs were seeded in the Transwell. OPCs were seeded into the Transwell at 3 (Day 3) or 6 days (Day 6) after the initiation of a neuron culture. Neurons were then cultured in the co-culture system for another 6 days. The length and number of neurites were quantified using the Day 3 neuron group after 6 days of co-culture with OPCs. Synaptogenesis was evaluated by synaptophysin and PSD-95 staining in the Day 6 neuron group after 6 days of co-culture with OPCs (Fig. 6A). Groups included neuron medium control, OPC medium control, OPC in OPC medium, and OPC in OPC medium plus DCC-siRNA. Successful knocking down of DCC in neurons was confirmed by immunostaining and RT-qPCR (Supplementary Fig. 4; NC 22.67 + 1.76, DCC-siRNA 8.00 + 1.15; *p* (NC vs DCC-siRNA) = 0.002). The western blot result further confirmed that DCC expression was blocked after DCC-siRNA transfection in the co-cultured neurons (Fig. 6F). Using the *in vitro* OPC culture, we were able to evaluate that Netrin-1 was expressed by OPC, and the conditioned media of OPC culture contained more Netrin-1 than control media (Fig. 6B; *p* (NM vs OS) = 0.031, *p* (OM vs OS) = 0.033; NM: neuron medium, OM: OPC medium, OS: OPC supernatant). Immunostaining results presented that OPC promoted neurite growth and synaptogenesis of neurons, which were eliminated by knocking down DCC in neurons (Fig. 6C-D). The results of Western blot were consistent with the immunostaining results in cultured neurons (Fig. 6E-F). These results supported that Netrin-1 secreted by OPCs signaled through its DCC receptor on neurons to promote neurite growth and synaptogenesis in cultured cells.

## DISCUSSION

Ischemic stroke is a major cause of death and disability in the world [33]. Stem cells are regarded as promising therapeutic candidates for the treatment of stroke, through various mechanisms, such as cell protection, anti-inflammation, angiogenesis and neurogenesis enhancement [34, 35]. Among all stem cells, OPCs have been reported to contribute to remyelination and demonstrated promising results for the treatment of demyelination disease in animal models. Given its potential of directly generating oligodendrocytes from OPCs, OPCs therapy is expected be a promising cell source over other type of stem cells. However, it remains challenging to obtain OPCs at high purity with adequate numbers [36]. By far induced pluripotent stem cell (iPSC) can be used to obtain OPCs *in vitro* successfully [37], yet uncertainty still remains as to the tumorigenicity when applying stem cell-derived OPCs to clinical studies [38]. In this paper, OPCs isolated from rats were used, given their relative abundance and ease for harvesting compared with mice OPCs [39]. We chose the shaking method to isolate the OPCs, which relied on the differential adherent properties of glia. During the culturing, microglia and astrocytes attach to the flask more efficiently than OPCs, which allows the collection of OPCs in the suspension after shaking flask. But the contamination of microglia and astrocytes is inevitable in this process. Microglia and astrocytes presented in the isolated OPCs might influence the transplantation effects on animals. For instance, microglia enabled to promote remyelination through clearing cell debris and secreting remyelination factors in myelin injury diseases, including multiple sclerosis, ischemic stroke, and Alzheimer disease [40].

We demonstrated that OPCs transplantation at 7 days after ischemic injury reduced brain atrophy and promoted functional recovery of ischemic stroke mice. After the subacute phase, treating ischemic stroke would have a higher chance of being translated into patients with extended treatment window [41]. Transplanted OPCs accumulated as clusters first at injected site and dispersed after 7 days of injection, which indicated that transplanted OPCs have the migration capacity. Moreover, transplanted OPCs survived at least for 14 days after injection, but disappeared at 9 weeks after injection. It is possible that additional strategies which enhance the survival and differentiation of OPCs would further improve the beneficial effects of OPCs transplantation. Many factors have been confirmed to support the OPC survival *in vitro*, such as insulin-like growth factors, neurotrophin-3, ciliary-neurotrophic factor, leukemia inhibitory factor and interleukin 6 [42]. The mechanisms of OPC differentiation have been extensively investigated, which will be helpful for the regulation of OPC differentiation and further applied to promote remyelination [43, 44].

Our results demonstrated that the differentiation of transplanted OPCs was limited in cerebral ischemic mice. It is plausible that trophic factors secreted by OPCs play a more important role than cell replacement in peri-ischemic region. In this study, we identified Netrin-1 and CXCL12 as important factors that were involved in the OPC related post-stroke recovery. Our present data demonstrated that OPC was a potential source of Netrin-1, and more importantly, CXCL12 might come from multiple sources, among which ECs acted as primary source *in vivo*. In addition to our findings, multiple other trophic factors had been estimated by other research groups. For example, it has been reported that OPCs supported BBB integrity through secreting soluble factor transforming growth factor beta 1 (TGF-β1). They observed OPCs attached to endothelial cells via basal lamina. Furthermore, knockdown of TGF-β1 in OPCs resulted in cerebral hemorrhage and the loss of BBB function in mice [45, 46]. Additionally, OPCs exhibited the capacity of promoting angiogenesis both in the postnatal white matter angiogenesis and after cerebral ischemia [45]. The hypovascularized white matter activated hypoxia-inducible factor induced the production of Wnt7a/7b from OPCs, which increased the proliferation and tube formation of endothelial cells [47]. Moreover, OPCs in the cortex shifted from parenchymal subtype to perivascular subtype, resulting in angiogenesis [46]. The BBB integrity and angiogenesis are pivotal in the brain ischemia, how the delayed OPCs transplantation influences these processes through the paracrine mechanism needs further investigations.

Our results further verified that OPCs transplantation enhanced endogenous oligodendrogenesis and promoted neurite growth and synaptogenesis. The current data did not rule out other mechanisms by which the transplanted OPCs affected the recovery after cerebral ischemia. However, the fact that co-administration of AMD3100 abolished many of the benefits of OPC transplantation supported that CXCL12/CXCR4 played key roles in the OPC-induced recovery. Our results revealed that CXCL12 was increased in the ischemic brain after OPCs transplantation. CXCL12 expression increased in the acute phase of cerebral ischemia and decreased after 7 days in MCAO mice [48]. It was further discovered that CXCL12 played a bi-phasic role after ischemic injury. Delayed CXCL12 gene therapy promoted neurogenesis and angiogenesis and enhanced functional recovery after stroke [49]. Therefore, OPCs transplantation provided an approach to maintain CXCL12 after the post-acute phase of cerebral ischemia. By binding with CXCR4, CXCL12 played important role in regulating the migration and differentiation of OPCs [50, 51]. We hence evaluated the effects of co-administration of AMD3100, as an antagonist of CXCR4, which could cross the BBB and a small but measurable amount was detected in the cerebrospinal fluid (CSF) [52]. Identical results were obtained in this study where CXCR4 expression in brain was successfully blocked through AMD3100 administration. It is noteworthy that CXCR4 is expressed by many cell types, including but not limited to OPCs [53]. In order to further understand the specific roles of CXCR4 on OPCs, future studies using transgenic mice with OPC-specific CXCR4 knock out would be necessary.

Interestingly, although co-administrated AMD3100 inhibited the ability of OPCs to promote oligodendrogenesis, it only partially eliminated the beneficial effects of OPCs on atrophy volume and functional performance. It indicated that other mechanisms were involved in the enhanced recovery brought by OPCs transplantation. In parallel to the oligodendrogenesis, endogenous neuronal plasticity is markedly activated and can be promoted by the paracrine mechanism in the brain after ischemic stroke [54, 55]. Among these trophic factors, Netrin-1 is a crucial axon guidance molecule that has attracted much interest [56]. It has been reported that Netrin-1 promoted axonal branching in the fetal cortex and enhanced synaptic regeneration of neurons [57, 58]. Additionally, in cerebral ischemic mice, the overexpression of Netrin-1 promoted axonal regeneration and synaptic formation through binding to its receptor, DCC [59]. DCC mediates axon attraction, while the other receptor of Netrin-1, UNC5 was involved in the axon repulsion [60]. In MCAO rats, upregulated Netrin-1 and DCC promote axonal regeneration and synaptic formation [59]. In this work, we investigated whether DCC mediated Netrin-1-induced neurite growth and synaptogenesis *in vitro*. In normal primary cultured neurons, neurite growth and synaptogenesis were enhanced by co-culturing with OPCs. However, in DCC-siRNA transfected neurons, such enhancement was diminished. Thus, we conclude that OPCs promoted neurite growth and synaptogenesis via Netrin-1/DCC.

As current clinical trials support the safety and promise of stem cell therapy for ischemic stroke, we as pre-clinical research scientists aim at continued investigation into the mechanisms of these treatment strategies. In the past decade, we have explored the co-administration of neural stem cell (NSC) with vascular progenitor cell (VPC) [4], comparing the delivery route of Mesenchymal stem cell (MSC) [61-63], engineering endothelial progenitor cell (EPC) with chemokines such as CXCL12 [11], and promoting NSC survival with optogenetic stimulation [64]. The results from these studies and the studies from other groups support that multiple mechanisms are involved in stem-cell based treatment, including but not limited to trophic paracrine mechanism, microenvironment modulation that reduces apoptosis and promotes regeneration, protection of BBB, modulation of glial activation and polarization that in-turn regulates neurovascular regeneration and remodeling, and finally small percentage of differentiation and integration into the neurovascular system. It is our hope that with the advancement of technology in stem cell manufacturing and engineering, multiple stem-cell based treatments could become available in the next decade. The field is also actively exploring cell-free treatment strategies using extracellular vesicles and microRNAs, leveraging the knowledge gained from stem-cell therapy research.

## Conclusion

This study demonstrated that delayed OPCs transplantation at 7 days after ischemic injury was beneficial for histological and functional outcomes in mice. Such benefits could be attributed to the enhanced endogenous oligodendrogenesis and the promoted neurite growth and synaptogenesis. Increased oligodendrogenesis was resulted from increased CXCL12. Augmented neurite growth and synaptogenesis were results of the interaction between Netrin-1 secreted by OPCs with its DCC receptor on neurons. The results from our work suggest that OPCs transplantation may be a promising treatment for ischemic stroke.

## Supplementary Materials

The Supplemenantry data can be found online at: www.aginganddisease.org/EN/10.14336/AD.202.0416.


