# Supplementary material for: Oligodendrocyte Precursor Cells Transplantation Improves Stroke Recovery via Oligodendrogenesis, Neurite Growth and Synaptogenesis
Source: Aging Dis. 2021 Dec 1;12(8):2096–112. doi: 10.14336/AD.2021.0416 (PMC8612617; doi:10.14336/AD.2021.0416)
Supplement: Supplementary file 1 [file AD-12-8-2096-s.pdf]

SUPPLEMENTARY DATA

Oligodendrocyte Precursor Cells Transplantation Improves Stroke Recovery via Oligodendrogenesis, Neurite Growth and Synaptogenesis

Wanlu Li[1,#], Tingting He[3,#], Rubing Shi[1], Yaying Song[2], Liping Wang[2], Zhijun Zhang[1], Yaohui Tang[1], Guo-Yuan Yang[1,2*], Yongting Wang[1*]

© 2021. Li W et al. Published online at http://www.aginganddisease.org/EN/10.14336/AD.2021.0416

SUPPLEMENTARY DATA

Supplementary Table 1. Primary antibodies

Antibody	Company	Catalog number	Diluted concentration
NG2	Millipore	AB5320	1:200
PDGFR-α	Santa Cruz	sc-338	1:50
GFAP	Millipore	AB5804	1:200
MBP	Abcam	ab7349	1:200
MAP2	Millipore	AB5622	1:200
Synaptophysin	Abcam	ab52636	1:100
Tuj1	Millipore	MAB1637	1:200
Netrin-1	R&D	AF128	1:200
DCC	Santa Cruz	sc-11437	1:200
BrdU	Santa Cruz	sc-56255	1:200
CXCL12	Abcam	ab25117	1:500
CXCR4	R&D	MAB21651	1:500
CXCR7	Proteintech	51024-1-ap	1:250
β-actin	Santa Cruz	sc-47778	1:1000
GAP43	Abcam	ab128005	1:1000
PSD 95	Millipore	MAB1596	1:1000
BDNF	Novus	NBP2-42215	1:500
bFGF	Millipore	05-118	1:1000

MBP, myelin basic protein; MAP2, microtubule-associated protein 2; DCC, deleted in colorectal carcinoma; BrdU, 5-Bromo-2′-deoxyuridine; CXCL12, C-X-C motif chemokine 12; CXCR4, C-X-C chemokine receptor type 4; CXCR7, C-X-C chemokine receptor type 7; PSD95, postsynaptic density protein 95; BDNF, brain-derived neurotrophic factor; bFGF, basic fibroblast growth factor.

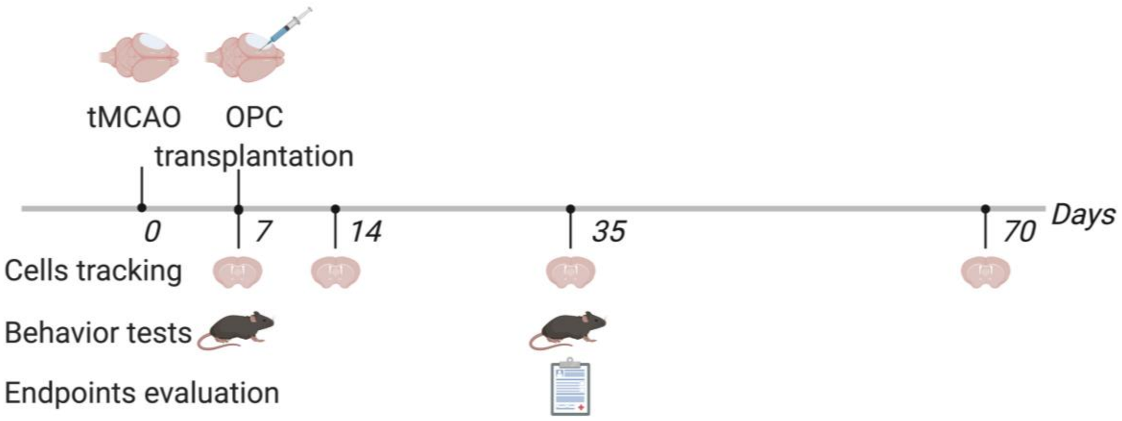

Supplementary Figure 1. Schematic of the experimental design, showing OPCs transplantation at 7 days after tMCAO, the evaluation of cell distribution at 7, 14, 35, and 70 days after tMCAO, the behavior tests at 7 and/or 35 days after tMCAO, and the endpoint detection at 35 days after tMCAO. tMCAO: transient middle cerebral artery occlusion.

© 2021. Li W et al. Published online at http://www.aginganddisease.org/EN/10.14336/AD.2021.0416

SUPPLEMENTARY DATA

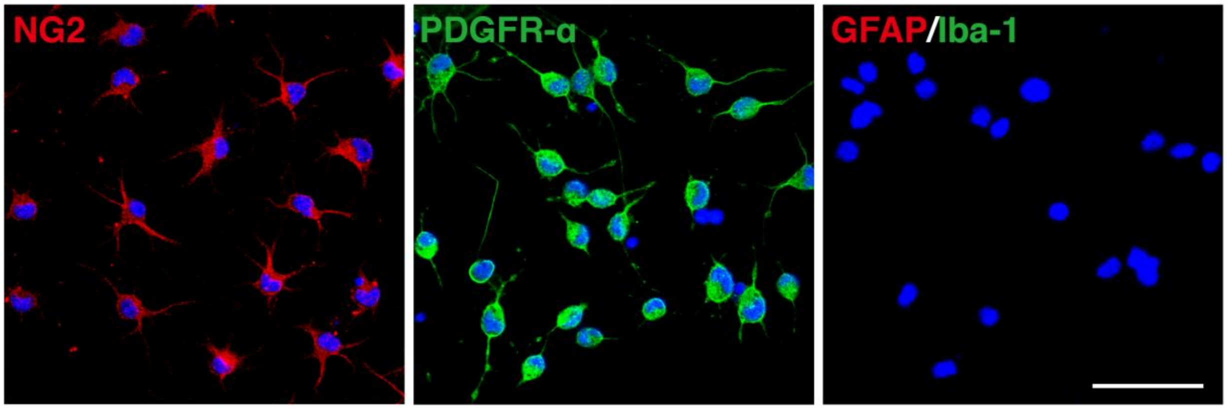

Supplementary Figure 2. The characterization of transplanted OPCs with NG2 (red), PDGFR-α (green), and GFAP with DAPI. Scale bar, 50 μm.

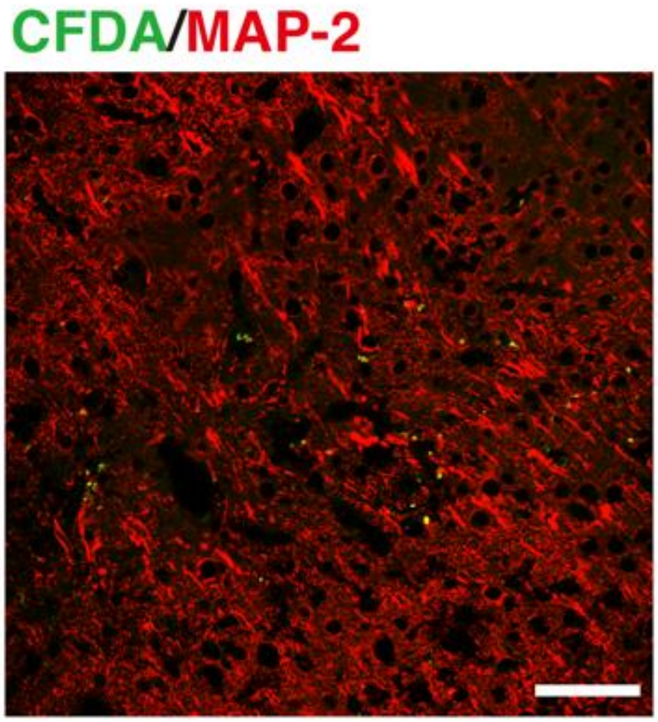

Supplementary Figure 3. Photomicrograph of CFDA SE (green) and MAP2 (red) in the periinfarct area at 35 days after tMCAO and 28 days after OPCs transplantation. Scale bar, 100 μm.

© 2021. Li W et al. Published online at http://www.aginganddisease.org/EN/10.14336/AD.2021.0416

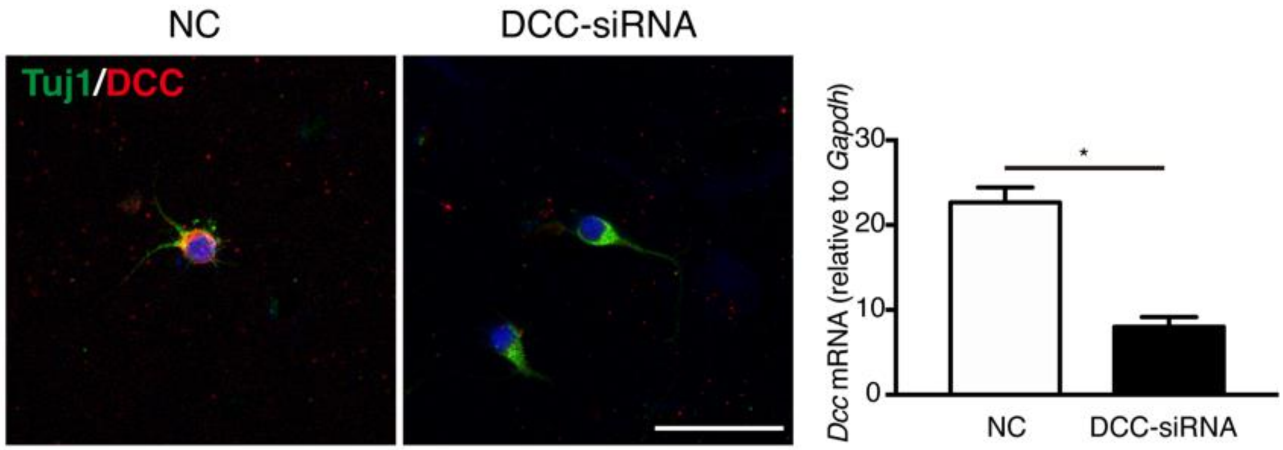

Supplementary Figure 4. DCC on cultured neurons was inhibited by DCC-siRNA. Images of Tuj1 (green) and DCC (red) in siRNA-transfected neurons. And the result of Dcc mRNA detection of siRNA-transfected neurons (n=3/group). Scale bar, 50μm. **p* <0.05. NC, negative control siRNA.

© 2021. Li W et al. Published online at http://www.aginganddisease.org/EN/10.14336/AD.2021.0416
